# A survey-based analysis of extended parental leave and work or educational absenteeism in Denmark due to respiratory syncytial virus in hospitalised infants

**DOI:** 10.1186/s12889-025-25926-9

**Published:** 2025-12-11

**Authors:** Nina Breinholt Stærke, Jan Håkon Rudolfsen, Jens Olsen, Mette Skovdal, Marie-Louise von Linstow

**Affiliations:** 1 Department of Infectious Diseases, Aarhus University Hospital, Aarhus, Denmark; 2 EY, Frederiksberg, Denmark; 3 Pfizer Denmark, Ballerup, Denmark; 4 Department of Pediatrics and Adolescent Medicine, Copenhagen University Hospital, Rigshospitalet, Copenhagen, Denmark

**Keywords:** Respiratory syncytial virus, RSV, Socioeconomic impact, Parental leave, Absenteeism, Healthcare policy, Denmark

## Abstract

**Background:**

Respiratory Syncytial Virus (RSV) is a leading cause of hospitalisation in infants worldwide. While the clinical implications of RSV are well-documented, the societal impact due to burden of illness absorbed by families remains underexplored. This study aims to quantify the burden on parents of infants aged 0–6 months hospitalised due to RSV infection during the 2022–2023 season in Denmark.

**Methods:**

A cross-sectional survey was conducted among parents of infants hospitalised with RSV. The survey included questions on the extension of parental leave, work and educational absenteeism, financial expenses related to medication, and the need for extended family support. The response rate was 29%, with 379 parents participating corresponding to 111 respondents.

**Results:**

Nearly half of the respondents (47.6%) reported extending their parental leave due to their child’s RSV hospitalisation, with the majority extending leave for 1–6 days. Work or educational absenteeism was reported by 24% of parents and 27% of their partners. Financially, 83% of parents faced additional expenses for medication. The study also found that 61% of families required support from family members, while few received assistance from local authorities. Based on the survey data, an estimated 2,484 to 10,416 workdays are lost due to RSV hospitalisations per season, indicating a significant societal cost.

**Conclusions:**

The socioeconomic impact of RSV hospitalisations extends beyond the immediate health implications for the infant. The findings reveal a substantial burden on parents, including extended leave, absenteeism, and financial costs. The study highlights the need to include burden on informal care givers when evaluating the burden of disease for infants and facilitate to reduce this burden.

**Supplementary Information:**

The online version contains supplementary material available at 10.1186/s12889-025-25926-9.

## Introduction

Respiratory Syncytial Virus (RSV) is a pervasive respiratory pathogen that inflicts a considerable health burden on infants and young children globally [1–3]. In infants and young children, especially those who are premature or have underlying health conditions, RSV may lead to lower respiratory tract infections which can cause severe illness with symptoms such as fever, apnoea, drowsiness, and respiratory distress [4]. Infants and young children with severe RSV infections may require hospitalisation and supportive care, which can include hydration, nasal suction and lubrication, oxygen therapy and in some cases, mechanical ventilation and intensive care [5, 6]. Worldwide, it is estimated that there are 3.6 million RSV-associated acute lower respiratory infection hospital admissions among 0–5 year-old children each year. Among these admissions it is estimated that 1.4 million were infants aged 0–6 months [3].

In Denmark, RSV infections are widespread and stands as a primary cause of hospital admissions for infants and young children particularly during its peak seasonal outbreaks usually occurring from late fall until early spring [4]. Notably, infants aged 0–6 months account for a significant proportion of the total RSV admissions among young children aged 0–5 years, with 59% being less than six months old [7]. Specifically, from 2019 to 2024, infants aged 0–6 months accounted for 1,000–1,500 RSV hospital admissions in each RSV season with most admissions occurring within a four-month peak period [8]. With approximately 60,000 births each year in Denmark [9], this corresponds to about 2% of all infants aged 0–6 months getting admitted to the hospital due to RSV. In Denmark, the direct costs attributable to RSV-related hospital admissions for infants aged 0–6 months have been estimated at EUR 4.3 million [10].

Moreover, the impact of RSV reaches far beyond the immediate burden on the healthcare system. Parents of infants with RSV face heightened emotional and psychological stress [11, 12] and are often compelled to take additional parental leave [13]. In Denmark, mothers being part of the labour force are entitled to 10 weeks of maternity leave after giving birth, while fathers/co-mothers have the right to 2 weeks after the birth. After the initial parental leave, each parent can take 32 weeks of leave, which can be extended up to 46 weeks [14]. The parental leave can be extended for the duration of hospital stay up to a maximum of three months [15]. Additionally, the co-parent may encounter work or educational absenteeism while caring for siblings of the admitted infant, inducing a short term productivity loss [16].

The exact extent of the indirect societal implications caused by RSV among infants in Denmark remains poorly understood. Consequently, this study explored the indirect consequences related to RSV hospitalisations among infants aged 0–6 months and the subsequent burden placed on the parents that may affect their ability to work and their need of extended parental leave in Denmark.

Understanding these issues are important for a comprehensive and full picture of the societal burden due to RSV in Denmark. The findings are intended to contribute to a more robust understanding of the indirect implications for parents caused by RSV hospital admissions among infants and to inform public health policy and guide resource allocation in the future.

## Methods

We sought to quantify the need for extended parental leave and extent of work or educational absenteeism for Danish parents who have navigated the challenges of RSV-related infant hospitalisation and illness through a nationwide survey.

### Study population

The target population for the study consisted of parents whose infants were hospitalised with RSV during the 2022–2023 RSV-season when the infant was between 0 and 6 months old.

**Fig. 1:**
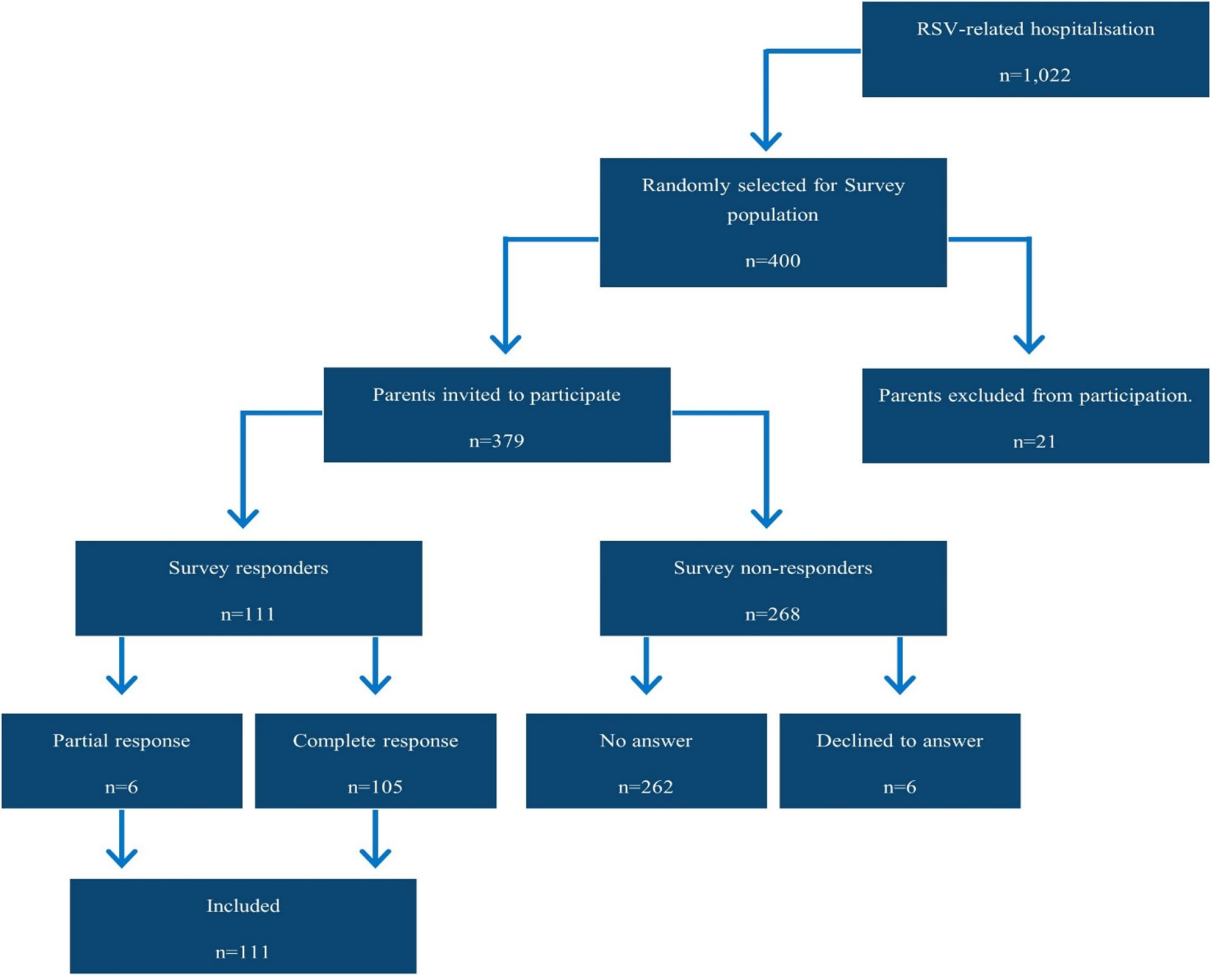
Survey response flowchart

Figure 1 presents the flowchart for identification of the survey population. 1,022 infants aged 0 to 6 months were identified as hospital admitted infants with RSV-related ICD-10 code registered as primary diagnosis codes (B97.4, J12.1, J20.5, J21.0) during the 2022–2023 RSV season. Of the 1,022 identified RSV admissions, 400 unique infants were selected at random to be the foundation for the survey population. The sample was compared with the not-selected population to ensure representativeness with respect to age at admission, sex and region of residence. Parents of the infants were identified in the Danish Central Person Register [17].

The parents were required to be residents of Denmark as of December 4, 2023, and at least 18 years old at that time. In cases where the infant was living with both parents, one parent was randomly selected to participate in the survey. To ensure responses from families with comparable living conditions, infants registered as not living with both parents at the time the survey was distributed had both parents were excluded.

### Data collection

The survey invitations and subsequent data collection in the study was facilitated by Statistics Denmark [18]. Invitations to participate were sent through Digital Post [19] (digital communication platform between public authorities and citizens in Denmark) and by mail for individuals exempted from Digital Post. Reminders were issued after 6 and 15 days to those who had not yet replied. The reminders were also delivered through Digital Post and by mail to individuals exempted from Digital Post.

The invitation letter included information about the background and purpose of the study, as well as details on data usage and data security. Additionally, respondents were informed that the survey was conducted by Statistics Denmark and EY Denmark for the purpose of this study. Data collection was carried out through a web survey from December 12, 2023, to January 3, 2024. An English translation of the survey is available in Supplementary 1.

### Survey content

The survey consisted of 28 questions and had an average completion time of 4 min (Supplementary 1). The survey included several filters, ensuring that only eligible respondents were prompted to answer specific questions based on their previous answers. The survey was rigorously tested in collaboration with Statistics Denmark to ensure that the questionnaire was unambiguous, easily understandable and that the response categories were exhaustive, conceptually clear, and mutually exclusive.

The survey included a series of questions regarding parental leave and any extensions resulting from the infant’s RSV hospitalisation, as well as the parents’ employment status and any work or educational absences incurred due to the infant’s illness. It also probed into the support received from grandparents or other relatives, assistance from public authorities, and expenditures incurred by the parents associated with the RSV hospitalisation.

Additionally, the survey collected information on family characteristics, including, family structure, such as the number of adults and siblings present in the household, age of siblings and inquired about any history of RSV infections in older siblings.

### Analysis

Data are presented for all eligible respondents who participated in the survey. This includes both respondents who completed the survey fully and respondents who only completed the survey partially. Due to confidentiality requirements set by Statistics Denmark, we must implement discretionary measures when processing survey questions with less than three responses to prevent the possibility of identifying individual respondents. Consequently, we could not provide detailed granularity for certain questions in the survey. The responses from the survey were applied to extrapolate the work absenteeism and loss of parental leave for the RSV population.

### Legal and ethics requirements

The study was conducted in accordance with legal and regulatory requirements, as well as with scientific purpose, value and rigour. It was aligned with widely recognised research protocols as described in the International Ethical Guidelines for Epidemiological Studies, issued by the Council for International Organizations of Medical Sciences (CIOMS) [20]. Ethical committee approval was not required for this study as it did not involve interventions or collection of or research on biological materials [21].

## Results

### Survey population

From the 400 identified RSV infants, 379 parents (mother or father) were invited to participate in the survey as 21 were excluded due to address confidentiality or reservation from participating in surveys. No parents were excluded from the survey on the basis of living alone with their infant at the time of distribution. A total of 105 completed the survey, while 6 completed the survey partially, meaning that a total of 111 responses were included in the analysis. This resulted in an overall response rate of 29% (Fig. 1). Comparison between the not-sampled population, the respondents and the non-respondents are presented in Table 1.

**Table 1:** Overview of infant characteristics in the not-sampled population, and the non-responders and responders in the sampled population

Characteristics of infants by group	Not Sampled, *N* = 622	Questionnaire, Non-respondents, *N* = 289^*1*^	Questionnaire, Respondents, *N* = 111^*1*^	*p*-value^2^
Sex				0.4
Male	352 (57%)	171 (59%)	58 (52%)	
Female	270 (43%)	118 (41%)	53 (48%)	
Region				0.7
North Jutland	75 (12%)	35 (12%)	15 (14%)	
Central Jutland	182 (29%)	84 (29%)	30 (26%)	
Southern Denmark	121 (19%)	62 (21%)	18 (16%)	
Capital	163 (26%)	77 (27%)	36 (34%)	
Zealand	81 (13%)	31 (11%)	12 (10%)	
Age at admission (month) ^*1*^	1.8 (1.08, 3.00)	1.92 (1.08, 3.48)	2.04 (0.96, 2.88)	0.6

^*1*^ n (%); Median (IQR)

^*2*^ Pearson’s Chi-squared test; Kruskal-Wallis rank sum test

Among the respondents, 75 out of 111 (68%) were mothers of infants who had been hospitalised with RSV (Table 2) and 105 out of 111 (95%) respondents reported living with the infant’s other parent at the time of hospitalisation. The infant’s median age at the time of admission is presented in Table 1 and the median length of stay was 3.5 days (IQR: 1, 6.3).

**Table 2:** Family characteristics at time of hospitalisation

	Response
Caregiver who responded to the questionnaire	*N* = 111
Mother	75 (68%)
Father	36 (32%)
Resided with the infant’s other caregiver at time of hospitalisation	*N* = 111
Yes	105 (95%)
No	6 * (5%)
Individuals aged ≥ 18 years of age in the household	*N* = 111
1	15 (14%)
2	89 (80%)
≥ 3	7 (6%)
Children aged ≤ 6 years of age in the household, including the infant who were hospitalised	*N* = 111
1	30 (27%)
2	67 (60%)
3	14 (13%)
Children aged 7–17 years of age in the household	*N* = 111
Yes (1 or more) **	17 (16%)
No	93 (84%)
Older siblings diagnosed with RSV within the last year	*N* = 86
Yes	18 (21%)
No	25 (29%)
Don’t know	43 (50%)

*Note: Number of responses are viewed as* ***N (%)***

* Were living together at the time of distribution of the questionnaire, but not at the time of hospitalisation, ** Response category pooled as not to enable identification of respondents

It was reported that 81 (73%) of the RSV cases had at least one sibling aged six years or younger, while 16% reported at least one sibling aged 7–17 years old. Furthermore, 21% of the respondents reported at least one older sibling diagnosed with RSV in the last year.

The majority of respondents (67%) were on parental leave at time of hospitalisation (Table 3). The employment rate among the caregivers (respondents and respondents’ partner) was approximately the same as that of the general population (84% versus 85%), and their annual income was also comparable (EUR 52,900 versus EUR 52,100).

A comparison of the respondents with the general population in the same age group (the respondents had a mean age of 34 years) revealed that a higher percentage of the respondents identified as ethnic Danish (87% compared to 73%).

**Table 3:** Caregiver characteristics at time of hospitalisation

	The respondent	The respondent’s partner
Individuals on parental leave	*N* = 110	*N* = 104
Yes	74 (67%)	40 (36%)
No	36 (33%)	64 (58%)
Occupation of caregivers who were on parental leave (before going on parental leave)	*N* = 73	*N* = 38
Employed	55 (75%)	33 (87%)
Unemployed	8 (11%)	< 3 *
Studying	7 (10%)	< 3 *
Other or Don’t know *	3 (4%)	< 3 *
Occupation of caregivers who were not on parental leave	*N* = 34	*N* = 64
Employed	28 (82%)	61 (95%)
Other or Don’t know *	6 (18%)	3 (5%)

Note: Number of responses are viewed as ***N (%)***

* Response category pooled or made discretionary as not to enable identification of respondents

### Extended parental leave and absenteeism from work or education

Of those who were on parental leave at the time of their infant’s RSV hospitalisation, 36 out of 73 (49%) of respondents’ and 14 out of 39 (36%) of respondents’ partners’ parental leave was extended due to the infant’s RSV hospitalisation (Table 4). Thus, in total 50 out of 112 parents (45%) had their parental leave extended. In 25 out of 36 (69%) cases, the respondents extended their parental leave by 1 to 6 days and in 11 out of 36 (31%) cases the respondents did so by 1 to 2 weeks.

**Table 4:** Extended parental leave and work or educational absenteeism due to their infants RSV disease

	The respondent	The respondent’s partner
Individuals whose parental leave was extended due to their infant’s RSV	*N* = 73	*N* = 39
Yes	36 (49%)	14 (36%)
No or Don’t know	37 (51%)	25 (64%)
Extension of parental leave	*N* = 36	*N* = 14
1–6 days	25 (69%)	-
1–2 weeks	11 (31%)	-
Less than 2 weeks *	-	14 (100%)
Individuals who were absent from their job or education due to their infant’s RSV	*N* = 105	*N*= 100
Yes	25 (24%)	27 (27%)
No	71 (68%)	69 (73%)
Not relevant	9 (9%)	4 (4%)
The length of absence from job or education	*N* = 25	*N* = 27
1–6 days	19 (76%)	20 (74%)
1 week or longer	6 (24%)	7 (26%)
Individuals that had expenses related to the infant’s RSV	*N* = 105	Not reported
EUR < 13	9 (9%)	−
EUR 13–66	40 (38%)	−
EUR 67–133	23 (22%)	−
EUR > 134	16 (15%)	−
No expenses	14 (13%)	−
Don’t know	3 (3%)	−

Note: Number of responses are viewed as ***N (%).*** Conversion 0.13 DKK per EUR

* Response category pooled, as there were too few responses in one of the categories to be reported separately without the risk of enabling identification of respondents

In the first 12 months of the infant’s life, 25 out of 105 (24%) respondents and 27 out of 100 (27%) of respondents’ partners were absent from their job or education due to their infant’s disease with RSV. Among the respondents, 19 out of 25 (76%) reported absences for less than one week, while 6 out of 25 (24%) had absences exceeding one week. Similarly, 20 out of 27 (74%) of the respondents’ partners had absences less than one week, and 7 out of 27 (26%) experienced absences longer than one week.

Furthermore, 88 out of 105 (83%) respondents reported that their infant’s disease resulted in direct costs related to medication. In 40 out of 105 (38%) instances, the costs ranged from EUR 13 to 66. In 23 out of 105 (22%) cases, the costs were between EUR 67 to 133, and in 16 out of 105 (15%) cases, the costs surpassed EUR 134.

### Extrapolation of survey findings

111 responded to the survey and 105 complete responses were received, of which 47.6% (50 parents) reported extended parental leave from 1 day up to 2 weeks, making the total duration of extended parental leave in the survey population between 116 and 497 days. This corresponded to approximately 1 to 5 days of extended parental leave per RSV hospitalisation.

Additionally, in the first 12 months of the infant’s life, 24% of the respondents and 27% of the respondents’ partners were absent from their job or education due to their infant’s disease with RSV, resulting in between 81 and 325 days of sick leave. This corresponds to approximately 0.8 to 3 days of absenteeism per RSV hospitalisation. In total, the 105 respondents of our study generated between 164 and 680 days of labour market absenteeism (parental leave adjusted to a five-day work week), due to sick leave or extended parental leave. The upper limit of the annual RSV admissions per year from 2019 to 2024 of 1,500 admissions among infants aged 0–6 months in Denmark [8] can be extrapolated to a loss of production in one RSV season ranging from 2,484 to 10,416 days. See Supplementary 2 for a further explanation of the calculations.

### Practical support to the families

Among the respondents, 64 out of 105 (61%) indicated that they had received varying levels of assistance from family members during their infant’s illness with RSV (Table 5). The majority received help for 0–20 h, with 24 out of 64 (38%) receiving less than 10 h, 15 out of 64 (23%) receiving help for 10–20 h, 8 out of 64 (13%) receiving help for 21–30 h, and 14 of out 64 (22%) receiving more than 30 h of assistance. Furthermore, 10 out of 105 (10%) respondents had also received professional help from the local authorities, most of which consisted of home visits from a nurse.

**Table 5:** Practical support to the families due to the child’s RSV disease

	Response
Individuals who received help from their family	*N* = 105
Yes	64 (61%)
No	41 (39%)
Hours of help from family	*N* = 64
Less than 10 h	24 (38%)
10–20 h	15 (23%)
21–30 h	8 (13%)
More than 30 h	14 (22%)
Don’t know	3 (5%)
Individuals who received help from the local authorities (e.g. visits from a nurse, additional care)	*N* = 105
Extra visits from home nurse	7 (7%)
Other help from local authorities *	3 (3%)
No	95 (90%)
Number of visits from the visiting nurse	*N* = 7
1	4 (57%)
2	3 (43%)

Note: Number of responses are viewed as *N (%)*. Response is viewed as ***N (%)***

* Response category pooled as not to enable identification of respondents

## Discussion

To the best of the authors’ knowledge, this study is the first to survey the implication of RSV hospitalisation in infants on extension of parental leave and work or educational absenteeism in Denmark.

We estimated that between 164 and 680 days of absenteeism were generated due to RSV disease in the surveyed population, corresponding to 2,484–10,416 annual days of absenteeism due to RSV hospitalisations in infants ˂6 months of age.

This estimate of forgone production may be a severe underestimation of what it should have been if every parent exercised their rights to extended parental leave. For parental leave to be extended in Denmark, parents must apply to the authorities, documenting the length of hospital stay [15]. The parental leave will then be extended accordingly. However, based on the survey results this right was only exercised in 47.6% of the cases. In other words, in 52.7% of the cases, parents forgo their right to extended parental leave, hence, having experienced a welfare loss of not having full parental leave. Therefore, the estimate of forgone production could have been even larger if all parents exercised their right to extended parental leave after the infants RSV hospitalisation.

This loss in utility and lost production does not include the added practical support from family members or local authorities. Nor does it include the out-of-pocket expenses related to the RSV episode. Moreover, the psychological stress experienced by parents dealing with their infant’s RSV can be overwhelming, and it is possible that the added application and documentation process associated with parental leave extension feels like too much for individuals in a vulnerable position. Faced with the immediate demands of caring for a sick child, the prospect of assembling paperwork and applications can seem daunting, leading parents to forgo their right to extended parental leave. To ease the psychological stress on parents during infant hospitalisations in general, a simplified and automatic system for extending parental leave could be implemented. This would allow parents to concentrate on their infant’s recovery without the added strain of navigating administrative processes.

All in all, RSV is an excellent case study to highlight one of the most discussed topics in evaluation of new preventions in recent years; how should burden on informal caregivers be included in evaluation of new medications? In Sweden, this topic was discussed extensively in a report by Karolinska Institute in 2022 [22], and a recent evaluation of policy in Norway recommended including lost production and informal caregivers to be included in an accompanying ‘societal perspective analysis’ when evaluating new treatments (as opposed to a health perspective as is currently applied) [23].

The results presented in this study highlights the importance of including the societal perspective when evaluating the burden of illness. Thus, RSV is an example where assessment and evaluation of new vaccines or treatments should include lost productivity to ensure a valid estimate of the full societal benefit. Parallels can for example be drawn to migraine or dementia, where lost production is a consequence of either own forgone production, or forgone production from those who provide informal care.

### Findings in other studies

Few studies have previously investigated the indirect effects of infant RSV hospitalisation for parents. A study by Hak et al. [24] found that 28% of parents in an European cohort (Spain, Scotland, England, Finland and the Netherlands) were absent from work due to RSV infections among infants (˂12 months of age) requiring medical attention (general practitioner, outpatient clinic, emergency department, or hospitalisation). In case of parental work absenteeism, the median duration was 4 days per RSV case [24]. Trautmannsberger et al. [25] found that among working parents in Germany, France, Italy, and Sweden whose children (˂24 months of age) were hospitalised for RSV, the average working time missed due to their child’s illness was 29 h. Furthermore, the study indicated that the job productivity among working parents during their child’s illness was ‘Very much’ or ‘Moderately’ impacted in 40% and 26% of cases, respectively, further underscoring the broader indirect impact of hospitalisation of young children due to RSV. The findings by Hak et al. [24] and Trautmannsberger et al. [25] closely resemble the findings of our study suggesting that our results on work absenteeism among parents due to RSV in infants in Denmark are consistent with other European nations.

### Strengths and limitations

Several robustness measures of the survey were implemented. We used national registries for participant recruitment, ensuring that only relevant individuals were invited. This recruitment strategy also enabled us to evaluate the representativeness of the sample, relative to the population. Our analysis confirmed that the respondents accurately reflected the demographic profile of parents who experienced infant RSV hospitalisations during the 2022/2023 season, thereby increasing the reliability of our findings. Another strength of our study was a low incidence of survey fatigue. This was evident by a completion rate of 105 out of 111 participants, suggesting that the survey was well-received and manageable for participants.

Despite these strengths, our study also faced limitations. Without a control group, we did not have a comparative baseline and it was challenging to determine whether the outcomes observed in the survey was directly attributed to RSV. This restricts our ability to draw definitive conclusions about the impact of RSV hospitalisations among infants. Furthermore, the sample size for certain questions was small, leading to an inherent uncertainty in the results. The limited sample size may introduce selection bias, as those who chose to respond may differ significantly from non-responders in ways that are critical to understanding the overall impact of the RSV hospitalisation in infants. The study also relies on only a few characteristics, and this restricts our ability to assess the demographic diversity of our respondents making it challenging to determine whether our findings are generalisable to the broader Danish population. However, it should be noted that the respondents were comparable with the general population in terms of employment and income. Recall bias was also a potential limitation. Despite only including parents whose infants were hospitalised in the previous RSV season, respondents were required to recall specific events related to their infant’s illness up to one year after they happened. The reliance on memory this far back in time may have affected the accuracy of the reported information. Another limitation is how the respondents may have interpreted question 4 in our questionnaire (see supplementary material). We acknowledge that some respondents may have answered this question as the number of people exclusive themselves that were living in the home. They might have answered that only 1 person above the age of 18 was living in the home when in fact 2 people above the age of 18 were living in the home. This could explain why almost 10% seem to have a co-parent under the age of 17, which would be high for Denmark.

Lastly, the use of ranges to report parental leave extensions may obscure the precise impact of RSV-related hospitalisations on individual leave durations. While ranges provide a general overview of trends and may reduce the risk of recall bias, they lack the specificity that could enhance understanding of the exact number of days parents needed to extend their leave. This limitation may hinder the ability to draw more detailed conclusions regarding the relationship between infant RSV-hospitalisation and the duration of extended parental leave.

## Conclusion

The study shows that RSV hospitalisations among infants aged 0–6 months often are associated with indirect consequences in terms of extended parental leave and increased absenteeism from work or education for parents. In addition, many parents are faced with the need of support from relatives or local authorities to manage the difficulties associated with their infant’s RSV illness.

## Supplementary Information

Supplementary Material 1.

## Data Availability

The data used is not publicly available and data cannot be shared due to regulations set up by Statistics Denmark. Requests to corresponding author can be made for aggregated statistics.
