# Supplementary material for: A survey-based analysis of extended parental leave and work or educational absenteeism in Denmark due to respiratory syncytial virus in hospitalised infants
Source: BMC Public Health. 2025 Dec 11;26:201. doi: 10.1186/s12889-025-25926-9 (PMC12801588; doi:10.1186/s12889-025-25926-9)
Supplement: Supplementary file 1 — Supplementary Material 1. [file 12889_2025_25926_MOESM1_ESM.docx]

# Supplementary 1: Survey

| Survey of parents of infants previously hospitalised with RS virus | | |
| --- | --- | --- |
|  |  | September 2023 |
|  |  |  |
| RED TEXT IS NOT DISPLAYED TO THE RESPONDENTS. | | |

**Intro**

Statistics Denmark and EY are currently conducting a study in Denmark of the consequences for infants, their parents, the healthcare system and society of infants under 6 months of age being hospitalised with respiratory syncytial virus (RS virus).

The study consists partly of a compilation of anonymised data on hospitalised infants from the Danish health registers and Statistics Denmark, as well as a questionnaire for randomly selected parents of infants that were hospitalised with RS virus from autumn 2022 to spring 2023.

The purpose of the questionnaire survey is to gather data on the consequences for the parents of the infant's disease. The questionnaire is being sent out to one parent, but it includes questions that concern both parents.

You have been selected as a parent of an infant that has been admitted to hospital with RS virus in the period from autumn 2022 to spring 2023, and you are hereby invited to answer the questionnaire.

Welcome to the survey about RS virus in infants. Start the form by clicking on the next page. Thank you for your help.

Q 1 Is it correct that you are the parent of a child that was hospitalised due to RS virus during autumn-spring 2022/2023?

- Yes
- No, my child had RS virus, but was not hospitalised (screen out)
- No, my child was hospitalised, but did not have RS virus (screen out)
- No, my child was not hospitalised and did not have RS virus (screen out)
- Don’t know
- Prefer not to say

When we write "your child" in the following, we mean the child that was hospitalised with RS virus in autumn-spring 2022/2023.

Q 2 Are you the child's mother or father?

- Mother
- Father
- Don’t know
- Prefer not to say

Q 3 Were you living with the child's other parent when the child was hospitalised?

- Yes
- No
- Don’t know
- Prefer not to say

Q 4 In total, how many people aged over 18 years, including yourself, were living in your home when your child was hospitalised?

- 1
- 2
- 3
- 4 or more
- Don’t know
- Prefer not to say

Q 5 In total, how many children aged under 7 years, including your hospitalised child, were living in your home when your child was hospitalised?

- 1
- 2
- 3
- 4 or more
- Don’t know
- Prefer not to say

Q 6 In total, how many children aged between 7 and 17 years were living in your home when your child was hospitalised?

- 0
- 1
- 2
- 3
- 4 or more
- Don’t know
- Prefer not to say

If 2 or more in Q 5 or 1 or more in Q 6

Q 7 Have any of your child's older siblings been infected with RS virus in the last year?

- Yes
- No
- Don’t know

Q 8 Were you on parental leave when your child was hospitalised?

- Yes
- No
- Don’t know
- Prefer not to say

If "Yes" to Q 8

Q 9 Was your parental leave extended as a consequence of your child's disease progression with RS virus?

- Yes
- No
- Don’t know
- Prefer not to say

If "Yes" to Q 9

Q 10 By how much was your parental leave extended as a consequence of your child's course of illness with RS virus?

- 1-6 days
- 1-2 weeks
- 2-3 weeks
- 3-4 weeks
- Longer than 4 weeks
- Don’t know
- Don't want to answer

If "Yes" to Q 3

Q 11 Was the child's other parent on parental leave when your child was hospitalised?

- Yes
- No
- Don’t know
- Prefer not to say

If "Yes" to Q 11

Q 12 Was the child's other parent's parental leave extended as a consequence of your child's course of illness with RS virus?

- Yes
- No
- Don’t know
- Prefer not to say

If "Yes" to Q 12

Q 13 By how much was the child's other parent's parental leave extended as a consequence of your child's course of illness with RS virus?

- 1-6 days
- 1-2 weeks
- 2-3 weeks
- 3-4 weeks
- Longer than 4 weeks
- Don’t know
- Prefer not to say

If "Yes" to Q 8

Q 14 When your child was hospitalised during your parental leave, what was your employment status?

- Working
- In education
- Available for work
- At home
- On sick leave (full or partial)
- In flexible employment, work trial, senior work scheme or similar
- On social security benefits, in early retirement or some other public support
- Don’t know
- Prefer not to say

If "No" to Q 8

Q 15 What was your employment status when your child was hospitalised?

- Working
- In education
- Available for work
- At home
- On sick leave (full or partial)
- In flexible employment, work trial, senior work scheme or similar
- On social security benefits, in early retirement or some other public support
- Don’t know
- Prefer not to say

If "Yes" to Q 11

Q 16 When your child was hospitalised, from what employment status was the child's other parent on parental leave?

- Working
- In education
- Available for work
- At home
- On sick leave (full or partial)
- In flexible employment, work trial, senior work scheme or similar
- On social security benefits, in early retirement or some other public support
- Don’t know
- Prefer not to say

If "No" to Q 11

Q 17 What was the child's other parent's employment status when your child was hospitalised?

- Working
- In education
- Available for work
- At home
- On sick leave (full or partial)
- In flexible employment, work trial, senior work scheme or similar
- On social security benefits, in early retirement or some other public support
- Don’t know
- Prefer not to say

**Q 18** Have you been absent from work or education in your child's first 12 months as a result of your child's course of illness with RS virus? Do not include planned parental leave.

- Yes, absent from work
- Yes, absent from education
- Yes, absent from both work and education
- Not relevant – was not in work or education during this period
- No
- Don’t know
- Prefer not to say

If "**Yes, absent from work" or "Yes, absent from both work and education" to Q 18**

**Q 19** For how long in total do you think you were absent from work in your child's first 12 months as a result of your child's course of illness with RS virus? Do not include planned parental leave.

- 1-6 days
- 1-2 weeks
- 2-3 weeks
- 3-4 weeks
- Longer than 4 weeks
- Don’t know
- Prefer not to say

If "**Yes, absent from education" or "Yes, absent from both work and education" to Q 18**

Q **20** For how long in total do you think you were absent from education in your child's first 12 months as a result of your child's course of illness with RS virus? Do not include planned parental leave.

- 1-6 days
- 1-2 weeks
- 2-3 weeks
- 3-4 weeks
- Longer than 4 weeks
- Don’t know
- Prefer not to say

If "Yes" to Q 3

**Q 21** Was your child's other parent absent from work or education in your child's first 12 months as a result of your child's course of illness with RS virus? Do not include planned parental leave.

- Yes, absent from work
- Yes, absent from education
- Yes, absent from both work and education
- Not relevant – was not in work or education during this period
- No
- Don’t know
- Prefer not to say

If "Yes, absent from work" or "Yes, absent from both work and education" to
Q **21**

Q **22** For how long in total do you think the child's other parent was absent from work in your child's first 12 months as a result of your child's course of illness with RS virus? Do not include planned parental leave.

- 1-6 days
- 1-2 weeks
- 2-3 weeks
- 3-4 weeks
- Longer than 4 weeks
- Don’t know
- Prefer not to say

If "Yes, absent from education" or "Yes, absent from both work and education" to
Q **21**

Q **23** For how long in total do you think the child's other parent was absent from education in your child's first 12 months as a result of your child's course of illness with RS virus? Do not include planned parental leave.

- 1-6 days
- 1-2 weeks
- 2-3 weeks
- 3-4 weeks
- Longer than 4 weeks
- Don’t know
- Prefer not to say

**Q 24** Have you had extra help from grandparents or other relatives in looking after siblings, cleaning, cooking etc. as a result of your child's admission to hospital with RS virus?

- Yes
- No
- Don’t know
- Prefer not to say

If "Yes" to **Q 24**

**Q 25** How many hours of help in total do you think you had while your child was hospitalised and immediately afterwards?

- Less than 10 hours
- 10 – 20 hours
- 21 – 30 hours
- More than 30 hours
- Don’t know

**Q 26** Have you had any extra help from the municipality where you were living while your child was hospitalised with RS virus and following discharge?

- Yes, extra visits from the visiting nurse
- Yes, extra care for the child's older siblings
- Yes, other help
- No
- Don’t know
- Prefer not to say

If "Yes, extra visits from the visiting nurse" to **Q 26**

**Q 27** How many extra visits did you have from the visiting nurse?

- 1 extra visit
- 2 extra visits
- 3 extra visits
- More than 3 extra visits
- Don’t know

**Q 28** Did you have any expenses as a result of your child's RS virus during the child's first 12 months (e.g. for medicine, saline drops, nasal aspirator, inhalers or similar)?

- Yes, less than DKK 100
- Yes, DKK 100 – 499
- Yes, DKK 500 – 999
- Yes, DKK 1,000 – 2,000
- Yes, more than DKK 2,000
- No
- Don’t know

**Screened out**We are sorry, but unfortunately you are not in the target group for this survey. The survey concerns only parents of children hospitalised due to RS virus in autumn-spring 2022/2023. Thank you for taking the time to respond. You can now close down the window, or you can click forward below and read more about surveys with Statistics Denmark.

**Questionnaire completed**That was the final question in the questionnaire. Thank you very much for your time and for taking part in the survey. If you have any comments about the questionnaire or the survey in general, you are welcome to write them down here. You can now close down the window, or you can click forward below and read more about surveys with Statistics Denmark.

# Supplementary 2: Calculations for extrapolation

| **Parental leave** | N | Conservative value | Max value | Sum conservative estimate* | Sum max estimate* |
| --- | --- | --- | --- | --- | --- |
| Respondents, reporting 1-6 days extended parental leave | 25 | 1 | 6 | **25** | **150** |
| Respondents, reporting 1-2 weeks extended parental leave | 11 | 7 | 14 | **77** | **151** |
| Partner, extended parental leave up to two weeks | 14 | 1 | 14 | **14** | **196** |
| SUM |  |  |  | **116** | **497** |

| **Sick leave** | N | Conservative value | Max value | Sum conservative estimate* | Sum max estimate* |
| --- | --- | --- | --- | --- | --- |
| Respondents, reporting 1-6 days sick leave | 19 | 1 | 5 | **19** | **95** |
| Respondents, reporting 1-2 weeks sick leave | 6 | 7 | 10 | **42** | **60** |
| Partner, sick leave 1-6 days | 20 | 1 | 5 | **20** | **100** |
| Partner sick leave 1-2 weeks | 7 | 7 | 10 | **7** | **70** |
| SUM |  |  |  | **81** | **325** |

*Sum conservative estimate = N*Conservative value

*Sum max estimate = N*Max value

| Sum, forgone production: | Parental leave + sick leave | Sum | Potential extension in survey population** | Absentee days per case** | Potential days absenteeism per RSV case** | Total RSV cases per year | Absentee days per year** | Potential absentee days per year** |
| --- | --- | --- | --- | --- | --- | --- | --- | --- |
| Conservative | 116+81 | **197** | 325 | 1.9 | 3.1 | 1500 | 2,814 | 4,639 |
| Max | 497 + 325 | **822** | 1,369 | 7.8 | 13 |  | 11,743 | 19,559 |

| Sum, forgone production. Parental leave adjusted for five day work week: | Parental leave + sick leave | Sum | Potential extension in survey population** | Absentee days per case** | Potential days absenteeism per RSV case** | Total RSV cases per year | Absentee days per year** | Potential absentee days per year** |
| --- | --- | --- | --- | --- | --- | --- | --- | --- |
| Conservative | 116*(5/7)+81 | **164** | 255 | 1.7 | 2.4 | 1500 | 2,484 | 3,644 |
| Max | 497*(5/7)+ 325 | **680** | 1,071 | 6.9 | 10.2 |  | 10,416 | 15,297 |

**Potential extension in survey population = sick leave + (parental leave/response rate)

**Absentee days per case = Sum/number of respondents

**Potential days absenteeism per RSV case = Potential extension in survey population/number of respondents

**Absentee days per year = Total RSV cases per year * Absentee days per case

**Potential absentee days per year = Total RSV cases per year * Potential days absenteeism per RSV case
